# Supplementary material for: Impact of COVID-19 on older adults with cancer and their caregivers’ cancer treatment experiences study: The ICE-OLD study
Source: PLoS One. 2023 Sep 20;18(9):e0291756. doi: 10.1371/journal.pone.0291756 (PMC10511107; doi:10.1371/journal.pone.0291756)
Supplement: S1 File — (DOCX) [file pone.0291756.s002.docx]

**Topic guide for older adults and caregivers with cancer**

Thank you for agreeing to participate in this study. The purpose of this study is to examine the impact of COVID-19 on cancer treatment plans (those considering treatment) and cancer treatment experiences (those receiving cancer treatment) of older adults aged 65 years and older. We will also examine the impact of COVID-19 on caregivers caring for older adults during the pandemic. You are being asked to participate so that we can better understand your experience considering/receiving treatment for cancer at this time *(direct to older adult)* and your experience as a caregiver at this time *(direct to caregiver)*.

You have indicated in the consent form that you wish to (audio-record/not audio-record) your interview. Do you still wish to continue with this?

*If the participants agreed to audio-recording:* The audio file will be destroyed immediately after transcribing the interview.

*If the participant does not agree to audio-recording:* Your responses will be written down by the interviewer. Using this method will mean that the interview will take longer to complete).

You can decide to take a break from the interview at any time, to stop the interview, or choose to not answer any questions that you are not comfortable with.

Are you both ready to proceed with the interview?

We would like to hear about your experiences with the care you considered / received for your cancer *(direct to older adult)* and with the care you provided your loved one during the COVID-19 pandemic *(direct to caregiver)*. Everything you say will be kept confidential. Do you have any questions before we start the interview?

| **Older Adult** | **Caregiver** |
| --- | --- |
| 1. Could you tell me when you were diagnosed with cancer and what cancer treatments you have received so far? | 1. How long have you been caring for older adults with cancer? What type(s) of support do you typically provide? |
| 1. Have you had a discussion with your oncologist about COVID-19? | 1. If you were supporting your loved one prior to COVID-19, how has COVID-19 impacted your ability to support your loved one? |
| 1. What was discussed? Have you discussed any changes to your cancer treatment plan as a result of the COVID-19 pandemic? | 1. Could you describe if and how you were involved in the cancer treatment decision-making of your loved one? |
| 1. How did virtual care impact your treatment decision-making process? | 1. What is the impact of COVID-19 on the cancer treatment of your loved one? |
| 1. What cancer treatments are you currently receiving? How is COVID-19 impacting your cancer treatment pan (e.g. transportation to the hospital etc.)? | 1. What are challenges in the current situation to deliver the best cancer care you see for your loved one? What is currently not going well? |
| 1. Do you have concerns about going for a blood test or for receiving services, such as chemotherapy or a physical examination, in a hospital or other community based service? | 1. Has your loved one requested any changes to their treatment plan due to COVID-19? |
| 1. Have you requested any changes to your treatment plan due to COVID-19? What have you requested and why? | 1. Have you discussed with your loved one any changes to their cancer treatment plans due to COVID-19? |
| 1. Can you describe your appointments with your oncology team? What is your experience meeting your team using virtual care? Can you describe any changes in appointment frequency? | 1. What changes were requested? Who requested them? |
| 1. Could you describe if you feel your relationship with your oncology team has changed and how? What are the positive and negative changes? | 1. How is your loved one receiving care for their cancer? What are challenges due to the changes in care you described? |
| 1. Due to the changes to cancer care can you describe any needs (e.g. X) that are currently unmet? Could you describe which ones and how? | 1. Could you describe if you feel that your relationship with your loved one has changed and how? What are the positive and negative changes? |
| 1. How is COVID-19 impacting your everyday life? (explore physical distancing and the impact on mental and physical health, isolation) | 1. Due to the changes to cancer care do you feel your loved one or you have any needs that are currently unmet? Could you describe which ones and how? |
| 1. How is the media reporting on COVID-19 impacting your experiences particularly the negative portrayal of older adults (e.g. statements like older adults should not get a ventilator if they need etc.)? | 1. How is COVID-19 impacting your loved one and your everyday life? |
| 1. What resources would you like to see developed/made available for older adults with cancer during this pandemic or for the next waves of the pandemic? | 1. How do you think the media reporting on COVID-19 is impacting your loved one’s experiences particularly the negative portrayal of older adults (e.g. statements like older adults should not get a ventilator if they need etc.)? |
|  | 1. What resources would you like to see developed/made available for older adults with cancer and their caregivers during this pandemic or for the next waves of the pandemic? |

Thank you. These were all of the questions I had.

*If the participants agree to be contacted for follow-up:* You indicated in the consent form that we may contact you if we have clarifying questions during the analyses. Is this still accurate?

*If the participants do not agree to be contacted for follow-up:* You indicated in the consent form that you do not want to be contacted if we have clarifying questions during the analyses. Is this still accurate?

Thank you both for your time!
